# Nutrients for Prevention of Macular Degeneration and Eye-Related Diseases

**DOI:** 10.3390/antiox8040085

**Published:** 2019-04-02

**Authors:** Hock Eng Khoo, Hui Suan Ng, Wai-Sum Yap, Henri Ji Hang Goh, Hip Seng Yim

**Affiliations:** 1 Department of Nutrition and Dietetics, Faculty of Medicine and Health Sciences, Universiti Putra Malaysia, UPM Serdang 43400, Selangor, Malaysia; hockeng_khoo@yahoo.com; 2 Department of Food Science and Nutrition, Faculty of Applied Sciences, UCSI University, No. 1, Jalan Menara Gading, UCSI Heights, Kuala Lumpur 56000, Malaysia; GrraceNg@ucsiuniversity.edu.my; 3 Department of Biotechnology, Faculty of Applied Sciences, UCSI University, No. 1, Jalan Menara Gading, UCSI Heights, Kuala Lumpur 56000, Malaysia; wsyap@ucsiuniversity.edu.my; 4 Derma Health (Australia) Pty. Ltd., 1/36, Hensbrook Loop, Forrestdale 6112, WA, Australia; henri_goh@dermahealth.com.au

**Keywords:** anthocyanin, dietary supplement, lutein, mineral, vitamin, zeaxanthin

## Abstract

The risk of macular degeneration can be reduced through the consumption of antioxidant-rich foods, supplements, and nutraceutical formulas. This review focuses on the antioxidants, vitamins, and minerals that have been reported for reducing the risk of macular degeneration and other eye-related diseases. Antioxidants including anthocyanins, carotenoids, flavonoids, and vitamins have been shown to reduce the risk of eye-related diseases. Anthocyanins extracted from berries are powerful antioxidants. Cyanidin, delphinidin, malvidin, pelargonidin, peonidin, and petunidin are anthocyanin aglycones detected in berries, currants, and other colored fruits and vegetables. β-Carotene, as well as xanthophyll lutein and zeaxanthin, have been reported to reduce the risk of macular degeneration. Flavonoids from plants help in the prevention of eye-related diseases through anti-inflammatory mechanisms. A combination of these antioxidants, vitamins, and minerals possess a synergistic effect on the prevention or risk reduction of macular degeneration. Formulas have been developed as dietary supplements to cater to the high demand from consumers and patients with eye problems. Many of the formulated dietary supplements that are sold in the market have been clinically proven for their efficacy to treat eye diseases. Although the bioactivities in the supplement capsules or tablets have been scientifically established for reducing risks of several diseases, which include macular degeneration and other eye-related diseases, knowledge on the right dosage, efficacy, and bioavailability of antioxidants, vitamins, and minerals is important for consumers. The information may help them make the best decision in choosing the right dietary supplements and nutraceuticals following the evidence-based recommended dosages and reference intakes for improving general health and preventing eye-related diseases. This review covers the potential causal factors involved in eye diseases, clinically proven treatments, and controversial findings on the antioxidants in the prevention of macular degeneration. Future studies should consider multiethnic and multicenter trials for eliminating potential bias in research.

## 1. Introduction

In recent years, several clinical trials and epidemiological studies have been conducted to evaluate the role of nutrients to improve or prevent visual loss in the elderly [1,2,3,4]. Many of these nutrients are strong antioxidants. Among the causes of visual loss worldwide, age-related macular degeneration (AMD) is the second most common cause of blindness after cataract in all regions, accounting for 15.4% (Western Europe) to 19.5% (Eastern Europe) of all blindness burden [5]. The rising prevalence of eye diseases causes a major social and economic burden to the country health care costs.

AMD is one of the age-related degenerative diseases, and it affects the macula which is responsible for high-acuity daylight vision in the central area of the retina [4]. The causes of AMD are multifactorial and include genetic predisposition, aging, and high oxidative stress [4]. Up to date, numerous studies suggest a positive association between the dietary micronutrients and decreases of progression in AMD and other eye-related diseases [1,2,4,6]. These studies have generated interest in micronutrients with antioxidant capabilities to prevent the oxidative damage involved in the development of degenerative eye diseases. Thus, micronutrients including antioxidants, vitamins, and minerals are attractive as promising strategies for preventative intervention.

No previous studies have produced a concise report on the right dosage and constituent ingredients that may help to improve conditions related to eye diseases and macular degeneration. Referring to the current situation, many patients are self-medicating with several types of pharmaceutical products, which are available over-the-counter without a prescription. Thus, researchers and health care professionals are responsible for advising their patients and consumers about the nutrients and supplements that could be beneficial.

In this review, we considered the extensively studied nutrients for the prevention and treatment of macular degeneration and eye-related diseases. These nutrients are anthocyanins, carotenoids (lutein, zeaxanthin, and β-carotene), vitamin A, vitamin C, vitamin E, zinc, and selenium [7,8]. Lutein and zeaxanthin are the most potent antioxidants for the prevention or reduction in the risk of AMD and other eye-related diseases. These xanthophylls aid in eye health and have been shown to reduce the risk of several eye-related complications [9,10,11,12]. Vitamins A, C, and E are the most effective vitamins for reducing the risk of macular degeneration [13]. However, only vitamin A plays an essential role in the human retinal pigment epithelial cells, whereas vitamins C and E are known to act as antioxidants. Besides these vitamins, minerals such as zinc and selenium have been shown to be linked with eye diseases. Selenium is also a strong antioxidant for eye protection.

The physiological functions of these nutrients, in relation to macular degeneration and eye-related complications, are discussed in this review. The synergistic effects and efficacies of the nutrients, as well as the pathways and mechanisms involving inflammation of the eyes, have been reported by many scientific studies. The absorption and bioavailability of nutrients are also influenced by several factors. This paper is a comprehensive review of the related nutrients for the prevention and treatment of macular degeneration and eye-related diseases.

## 2. Antioxidant Nutrients

### 2.1. Anthocyanins

**Figure 1:**
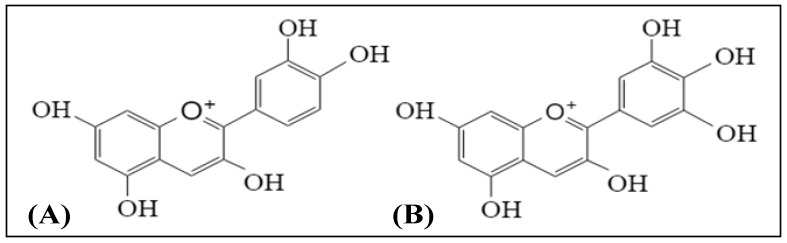
Chemical structures of (**A**) cyanidin and (**B**) delphinidin.

Anthocyanins are the phytochemicals belonging to the phenolic group. These compounds have chemical structures similar to flavonoids, with a positive charge on the C-ring of the flavylium backbone [14]. Anthocyanins are red–purple pigments found in plants, they are strong antioxidants and have been reported to be the major components in the red, blue, and purple coloring of flowers, fruits, and vegetables [15]. Berries are colored fruits that have high anthocyanins content. Berries, such as blueberry, bilberry, blackcurrant, strawberry, and wolfberry (better known as ‘goji berry’), are rich in anthocyanins. The berries also contain water-soluble flavonoid pigments—in part lending to the red, purple, and blue coloring of fruits and flowers—that act as potent antioxidants [16]. Among the berries, bilberry (*Vaccinium myrtillus*) extracts have been used as nutritional supplements [17,18]. Literature shows that bilberry extracts contained cyanidin and delphinidin as the major anthocyanin aglycones [19]. Among the six major, common anthocyanidins, cyanidin and delphinidin are abundantly found in most of the red to purple colored plants. The chemical structures of cyanidin and delphinidin are shown in Figure 1.

Anthocyanins are known to have antioxidant and anti-inflammatory properties, which may reverse oxidative stress and possibly improve certain diseases. These compounds stabilize free radicals by its hydrogen donating ability [20]. Anthocyanins have been shown to promote regeneration and synthesis of rhodopsin [21] to protect retinal from overexposure to visible light [22] and exposure to irradiation [23], as well as to improve vision and increase the supply of blood to the retina [24]. This is also evidenced by an animal study which demonstrated that the subjects’ eye, brain, and liver accumulated anthocyanins at week-4 of the blueberries–feed diet. The result showed that oral intakes of anthocyanins from natural sources (blueberries) provide the potential ocular protective benefits [25]. However, with the limitation of current laboratory level, one should be aware of its precise dosage and efficacy, and the potential toxicity and long-term side effects of the anthocyanins, especially when it comes from synthetic sources. The beneficial effects of anthocyanins in the prevention or risk reduction of AMD and other eye-related diseases are presented in Table 1. On the other hand, supplementation of 60 mg anthocyanins daily to the overweight and obese postmenopausal women (body mass index of 25–33 kg/m^2^) for eight months had no effect on macular pigment optical density [26].

### 2.2. Xanthophylls

**Figure 2:**
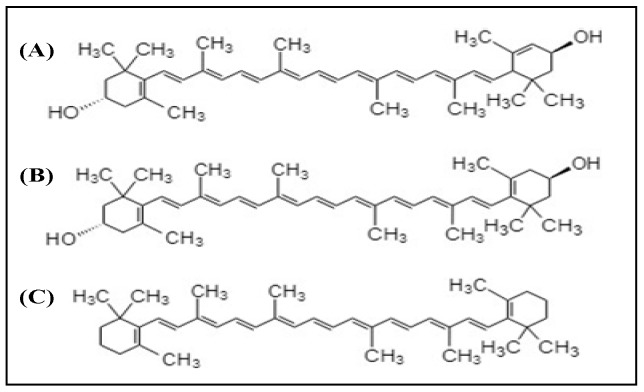
Chemical structures of (**A**) lutein, (**B**) zeaxanthin, and (**C**) β-carotene.

Xanthophylls are the compounds belonging to the carotenoid group. Lutein and zeaxanthin are the two dietary carotenoid xanthophylls. The structures of these compounds are shown in Figure 2. Among the carotenoids, xanthophylls have no pro-vitamin A activity besides β-carotene. These compounds concentrated in the macula and are therefore known as a macular pigment, found in the human retina [27]. Lutein and zeaxanthin are the xanthophylls that act in the biological systems as (i) important structural molecules in cell membranes, (ii) short wavelength light filters, and (iii) keepers of the redox balance [28]. However, the human body is not able to synthesize both lutein and zeaxanthin. For that reason, it must be obtained from the diet. The foods that have lutein and zeaxanthin content are green leafy vegetables and fruits such as kale, avocado, and maize. 

As shown in Table 1, xanthophylls including lutein and zeaxanthin, as well as β-carotene, are known to have protective effects against eye diseases and macular degeneration. Ma and Lin [29] also summarized the findings from several studies related to the protective effects of lutein and zeaxanthin on eye health, which included AMD, cataract, and retinitis pigmentosa. In this review, we summarized more recent studies related to serum concentrations of xanthophylls and the association with the risk of AMD and other eye-related complications.

Literature shows that the levels of lutein and zeaxanthin in plasma are associated with the decreased risk of macular disease such as AMD. A high intake of xanthophyll-containing foods has been attributed to the elevated levels of plasma lutein and zeaxanthin. As shown in the literature, short-term consumption of xanthophyll-rich foods, such as cooked egg yolk, vegetables, and spirulina, in the feeding group significantly increased plasma concentrations of the xanthophylls compared to the control group [11,30]. Therefore, the elevated levels of plasma lutein and zeaxanthin helped to prevent AMD in the elderly [9]. Table 1 shows evidence of the protective effect of lutein and zeaxanthin against macular diseases [10,11,12,30,31]. However, there exists no report or recommendation for the optimal dose of these xanthophylls for treatment and prevention of macular degeneration and eye-related disease [32].

There are many factors affecting the degeneration of macular pigment. Lifestyle and dietary factors are important to be taken into consideration for prevention or slowing the progression of early AMD. Poor lifestyle choices such as physically inactive, poor diet, and smoking increase the risk of AMD. A previous study shows that inflammation of macular pigment among heavy smokers was higher than light or non-smokers [33]. The finding showed that serum concentrations of lutein and zeaxanthin are essential for smokers because they have lower macular pigment optical density than non-smokers. Dietary intakes of lutein and zeaxanthin also differ with age, sex, and ethnicity. Johnson et al. [34] suggested that intake of lutein is recommended to be higher than zeaxanthin among all age groups. There is still no recommended daily intake for lutein and zeaxanthin, however, the randomized, double-blind, placebo-controlled human study showed health benefits at a daily intake of 10 mg of lutein and 2 mg of zeaxanthin [10].

Human intervention studies demonstrate that lutein and zeaxanthin supplementations improved visual performances, such as contrast sensitivity, glare tolerance, and photo stress recovery [10,29,35]. The result obtained from Age-Related Eye Disease Study 2 (AREDS 2) shows the beneficial effect of lutein and zeaxanthin supplementation related to AMD [36]. Based on scientific evidence (Table 1), β-carotene, lutein, and zeaxanthin are potent nutrients for reducing the risk of macular degeneration and eye-related diseases. On the contrary, one study reported that supplementation of 6 mg lutein and 2 mg zeaxanthin daily to postmenopausal women for eight months had no effect on the macular pigment optical density [24]. Owing to the fact that no specific doses have been prompted for the intakes of β-carotene, lutein, and zeaxanthin, policymakers and health care professionals should establish the dietary intake values for these carotenoids to encourage the public to increase the consumption of lutein-containing foods.

## 3. Vitamins

Vitamin A is closely related to its by-products, carotenoids, and plays several roles in the human body. Vitamin A intake and blood levels have also been examined for their roles in retinal health. Epidemiological data from the National Health and Nutrition Examination Survey (NHANES I) showed that those who consumed increased amounts of fruits and vegetables rich in vitamin A have a decreased risk for any stage of AMD [39]. The report summarized the protective effect of vitamin A with AMD.

Lately, a prospective population-based cohort also showed that long-term intake of fruits and vegetables that have provitamin A carotenoid further reduced risk of AMD [40]. On the contrary, several studies failed to show a significant association between increased dietary intake of vitamin A and reduced risk of macular degeneration (Table 2). Epidemiological studies showed no association between dietary intake of vitamin A and reduced risk of AMD [37,41]. With these conflicting results, more studies are needed to investigate the association between vitamin A and AMD.

Vitamin C (ascorbic acid) is an effective antioxidant that protects proteins, lipids, carbohydrates, and nucleic acids from free radicals and reactive oxygen species (ROS) damage [42]. Several fruits and vegetables are good sources of vitamin C [3]. Due to vitamin C being a strong antioxidant, it is beneficial to the human eye and helps to prevent eye-related diseases. The beneficial effect of vitamin C against eye diseases is supported by a study conducted by SanGiovanni et al. [43]. The finding revealed a reduced likelihood of neovascular AMD (abnormal blood vessels growing underneath the retina) in subjects reporting high intakes of β-carotene, vitamin C, and vitamin E. In addition, the results obtained from a meta-analysis also showed that the pooled odds ratio of vitamin C supplements was 1.11 (0.84 to 1.46), in reference to early AMD [38]. On the other hand, Christen et al. [44] reported that users of vitamin C as a dietary supplement had a higher relative risk (1.03) of exudative macular degeneration than the users of vitamin E and multivitamin supplements (relative risk of ≤0.9) but it was not statistically significant. However, the enhancement of AMD by vitamin C supplementation is not related to the patients’ genotype [45].

A recent Cochrane analysis conducted by Evans and Lawrenson [46] showed the controversial result that there was no significant association between vitamin C and primary prevention of AMD. The result was similar to the report published by the Eye Disease Case-Control Study Group [47] and Delcourt et al. [40], which showed no significant association between vitamin C intake and AMD. The no association between dietary vitamin C intake and reduced risk of AMD is further supported by Seddon et al. [39]. Another subsequent study showed no significant effects for vitamin C supplementation between the treatment group and placebo during the 10-year follow-up study [30]. On the whole, evidence from these studies shows no consistent relationship between dietary vitamin C intake and reduced risk for AMD. Therefore, further investigation is needed to be performed at a larger scale.

Vitamin E exists in four common forms in nature, namely, α-tocopherol, β-tocopherol, δ-tocopherol, and γ-tocopherol. It is an essential micronutrient and efficient antioxidant that scavenges free radicals. Deprivation of vitamin E could lead to lipofuscin accumulation [48], retinal damage [49], and loss of photoreceptors [50]. Due to vitamin E being a fat-soluble vitamin, it plays an important role in fatty acid metabolism. Vitamin E is involved in the desaturation of PUFAs via the microsomal electron transport chain [51]. In the human body, α-tocopherol has been shown to be the most abundant in both plasma and retinal tissue [52,53]. Thus, studies suggested that increased dietary levels of vitamin E have been correlated with increased concentrations in the retina, and epidemiologic studies also suggest a beneficial effect of vitamin E for fighting the progression of AMD [54].

On the other hand, supplementation of vitamin E as high as 500 IU daily in a randomized controlled trial did not show a positive outcome in preventing the development and progression of AMD [55]. Some other previous studies also reported that supplementation of vitamin E individually, or with a combination of vitamins A and C, did not significantly reduce the risk of AMD (Table 2), however, subjects with the high levels of plasma carotenoids had significantly lower risk than the others [56]. Back in the 90s, the Pathologies Oculaires Liées á l’Age (POLA) study in France also reported no significant association between vitamin E intake and AMD [40]. On top of AMD, vitamin E supplementation (400 IU) showed no protective effect against cataract in older subjects [57]. Based on past studies, a high dosage of vitamin E supplementation may not necessarily benefit eye health. However, the antioxidative protective effect of vitamin E could not be easily ruled out. More large-scale studies need to be carried out to ascertain its effect on preventing AMD in humans.

## 4. Minerals

Zinc is a co-factor of many metabolically active enzymes within the eye and essential for many physiological processes including immunity, reproduction, and neuronal development [62,63]. Zinc is found in ocular tissue, particularly the retina [64], which is the reason that zinc supplementation may aid retinal health. A moderate amount of zinc supplementation helps to protect the retina—this is because zinc toxicity may occur at a higher level of zinc intake. Also, the elderly may have an increased risk of vision loss from AMD due to their higher risk of zinc deficiency.

Aoki et al. [58] reported that a high dose of zinc (80 mg) alone, which obtained through supplementation, reduced the risk of progression of neovascular AMD in the AREDS study. Dietary self-administration of zinc (200 mg) daily for up to 24 months had also proven to significantly reduce the visual loss compared to the placebo group [59]. A randomized, controlled clinical trial by Newsome evaluating a novel zinc, monocysteine, for the treatment and prevention of AMD showed significant improvements in visual acuity and contrast sensitivity, as well as a shortened macular light flash recovery time, in the treatment group at the third and sixth months of the trials compared to the placebo group [60].

On the contrary, the AREDS [13] showed that dietary intake of zinc had no significant differences for all the secondary outcomes between the treatment group and placebo. Another study also reported negative findings for zinc versus AMD [61]. According to the systematic review conducted by Vishwanathan et al. [65], the authors stated that zinc intake for the prevention of AMD was inconclusive due to the inconsistency of the findings reported by the cohort studies. They concluded that zinc treatment might be effective in preventing progression to advanced AMD. The review also concluded that zinc supplementation alone might not be sufficient to produce clinically significant changes in visual acuity. In addition to these findings, Assel et al. [66] reported thatnone of the evidence to support the reduced risk of AMD by zinc supplementation was related to patients’ genotype. Klein and co-workers [45] reported, however, that the CC genotype of the patients could be a factor for the ineffectiveness of zinc supplementation in reducing the risk of AMD.

Selenium is well-known as an antioxidative agent, it is a trace element found in several enzymes of the human body. In the past years, a few studies have been focused on the effect of selenium in reducing the risk of AMD. Selenium-dependent glutathione peroxidase plays an important role in the protection against oxidative damage to membrane lipids [51,67]. Glutathione peroxidase functions as a mediator to reduce hydrogen peroxide and other possible hydroperoxides that present inside the cell. It is also hypothesized to be able to protect the macula against oxidative damage. However, these results have not been convincing and were inconclusive [47,68].

A low intake of dietary selenium is known to cause a reduction of total polyunsaturated fatty acids (PUFAs) in the retinal pigment epithelium and retinal rod outer segments of laboratory animals [69]. The fact is that selenium, together with vitamin E, is associated with fatty acid metabolism [51]. Due to the lack of case-control studies, the protective role of selenium and its enzymes in AMD remains unclear. Further studies warrant the efficacy of selenium for eye protection and the prevention of AMD and eye-related complications.

## 5. Absorption and Bioavailability of Nutrients and Antioxidants

Bioavailability is defined as the proportion of any ingested substance or nutrient that absorbed and entered the circulation system for normal physiological functions [70]. It differs from bioaccessibility, which is the quantity of the ingested nutrient that is available for absorption [71]. It is useful to be able to predict the bioavailability of a specific nutrient asnutrient or antioxidant absorption is closely related to the bioavailability of the specific nutrient or antioxidant. A nutrient that is highly bioavailable has a high absorption rate.

Many factors affect nutrient and antioxidant absorption such as stress, alcohol consumption, caffeine and drug intakes, and exercise. Absorption of nutrients and antioxidants is also dependent on the therapeutic doses. As reported in the literature, the increased amount of antioxidant intake reduced the absorption efficiency of antioxidants. For example, the absorption efficiency of lycopene was higher during the low dose intake than the high dose intake [72]. Another study also showed that an equal dose of zeaxanthin had a lower absorption than lutein [73]. In addition, heat stress could reduce nutrient absorption, thus reducing the absorption efficiency [74].

The other factor that affects nutrient absorption is a dietary factor, which is the key bioavailability factor. Nutrient absorption is known to be assisted by the intake of fats and oils. Olive oil has been shown to improve the lutein absorption and its bioavailability in mice with a lutein-deficiency through modification of the activity of intestinal triacylglycerol lipase [75]. Conversely, the literature shows that butter and palm oil with highly saturated fats enhanced the bioavailability of xanthophylls compared with the high PUFA oils [76]. The ingested amino acid ligands including histidine and cysteine are the dietary components that promote zinc absorption [77], whereas organic acids, such as ascorbic acid and citric acid, help to improve iron absorption [70].

In fact, nutrients from animal sources are more bioavailable than that of plant origin. Many intervention studies showed that zeaxanthin from egg yolk is more bioavailable than many other zeaxanthin-containing foods and dietary supplements [28,56]. Therefore, the consumption of egg yolk is able to reduce the risk of AMD [78]. The mechanism behind this observation is that animal products contain a higher amount of saturated fatty acids than plant-based foods. Besides these saturated fats, intestinal fatty acid binding protein is also hypothesized to be the main contributor to the increased absorption of nutrients from non-plant-based foods. This fatty acid binding protein is initially hypothesized to be essential for the absorption of dietary nutrient due to its ability to bind lipid ligands [79]. Also, meats and eggs have a low level of long-chain PUFAs. These unsaturated fatty acids have a higher affinity for the fatty acid binding protein to transport the selected nutrient into the cells than the saturated fatty acids [80]. In addition to the absorption of xanthophylls, minerals found in animal products have higher bioavailability than that those found in plants [81]. This is because plant-based foods contain a large concentration of antinutrients such as phytates and oxalates.

Bioavailability varies among different nutrients. A previous study showed that the bioavailability of lutein from vegetables is five times higher than β-carotene [82]. Also, the amount of fat in food affects the bioavailability of lutein. A higher amount of fat consumed together with lutein supplement by healthy subjectshas beenattributed to the increased intestinal uptake of lutein [83]. Therefore, consumption of lutein-rich foods can help in the prevention of AMD. Conversely, increased intake of lutein via supplementation is found to inhibit the absorption of β-carotene [84]. Higher absorption of lutein and zeaxanthin through the intake of these xanthophyll-containing vegetables is probably due to the chemical components in olive oil compared to the other types of vegetable oils [85].

## 6. Adverse Effects of Antioxidants

Scientifically, many nutrients obtained from foods are potent antioxidants. Some of these naturally occurring antioxidants such as anthocyanins and carotenoids have strong antioxidant abilities. Although these antioxidants are naturally occurring compounds, they might be nephrotoxic due to overconsumption. Synthetic antioxidants could also pose potential adverse health effects. Therefore, the right dose of antioxidant in dietary intake is key in the prevention of many diseases such as eye-related diseases. Hence, the intake of large doses of certain antioxidants or overdoses could be harmful and increase the risk of inflammatory disease such as cancer [86,87]. Therefore, a guide to antioxidant intake is essential for prevention of diseases and maintaining good health without adverse health effect or toxicity issues. In this review, tolerable upper intake levels (ULs) [88] and recommended daily intakes of vitamins and minerals for the prevention of macular degeneration and eye-related complications are shown in Table 3.

Among the phytochemicals, anthocyanins and xanthophylls pose no adverse health effect to the human body. Based on the literature search, there is no report of toxicity effects associated with the chronic consumption of anthocyanins [89] and xanthophylls [90]. Literature reports on the toxic effect of lutein and zeaxanthin are optimistic and showed no adverse effect due to these xanthophylls being obtained from natural sources. Short-term intakes of lutein and zeaxanthin supplements of up to 40 mg daily by patients with retinal degeneration showed no toxicity effect [91]. A review article also reported that no long-term adverse health effect on dietary intakes of lutein and zeaxanthin, as well as no in vitro mutagenic and cytotoxic activities, were observed [84].

An adequate amount of β-carotene helps to protect our body against inflammation and several chronic diseases. β-carotene is not toxic to the human body besides the skin yellowing with increased consumption of carotenoids from plants [92,93]. As reported by the AREDS research group, supplementation of β-carotene to smokers increased the risk of lung cancer; vitamin E supplementation to the AREDS’s participants also increased the risk of prostate cancer [13]. In addition to β-carotene, lycopene is a strong antioxidant that has an anti-inflammatory effect [94]. Although lycopene is not toxic, nor has any lycopene toxicity been reported [95], it has no provitamin A activity compared to β-carotene [94].

Vitamin A is an essential nutrient for the human eye. It is toxic if consumed in a large dose. Among the toxicity studies on vitamin A supplementation, most studies have been focused on acute toxicity using animal models [96]. The signs and symptoms of vitamin A toxicity are abdominal pain, anorexia, blurred vision, drowsiness, headache, hypercalcemia, irritability, muscle weakness, nausea and vomiting, peripheral neuritis, and skin desquamation [97]. Due to the toxic effects of vitamin A if overdosed, provitamin A carotenoid supplements are the substitutes for vitamin A in solving problems related to vitamin A deficiency. Provitamin A carotenoids supplementation is a safer way, and these carotenoids are the strong antioxidants for the prevention of many diseases [98]. On the contrary, Chew et al. [36] revealed that carotenoids, such as lutein and zeaxanthin, are the better choice for preventing or reducing the risk of developing AMD compared to β-carotene.

Although β-carotene has provitamin activity, no data is available for the UL of this compound because the figures reported in the literature are insufficient [99]. Among the vitamins, vitamin C has the lowest toxicity level. However, overconsumption of vitamin C may affect gastrointestinal [100] and renal function [101]. Literature shows that intake of vitamin C at higher doses 1000–5000 mg/day for healthy adults increased risk for renal stones, renal tubular disease, and oxaluria [88,93]. The recommendation of vitamin E allowance is dependent on the PUFA intake. Due to vitamin E being a fat-soluble vitamin, an increase in fat consumption might increase intake of vitamin E. Therefore, there is a high risk of vitamin E toxicity. Vitamin E toxicity is related to the long-term intake of a vitamin E supplement at a high dose. The literature showed that supplementation of vitamin E to experimental rats at either high dose or long-term supplementation caused minor physiological changes to the rats [93].

The toxicity cases of minerals including zinc and selenium are less reported in the literature compared with other minerals, such as copper and iron, as well as heavy metals. Findings from studies on zinc deficiency and adequacy have been reported in the literature [102]. Zinc toxicity is related tocongenital abnormality, stunning, neuropsychological impairments, and other clinical symptoms [103]. A case report reveals that acute overdose of zinc through oral intake of zinc gluconate (4 g) caused severe nausea and vomiting within 30 min of ingestion [104]. Experimental human subjects who were supplemented with zinc (440 mg zinc sulfate) daily for five weeks had a 25% reduction in high-density lipoprotein level [105]. Therefore, excessive intake of zinc is hazardous. Due to the seriousness of zinc toxicity, we report the UL of zinc intake as shown in Table 3. The UL of daily zinc intake should be 25 mg or below for a healthy adult.

Selenium is toxic to the human body if overconsumed. It is also a potent antioxidant if ingested in moderate doses. Vinceti et al. [106] reported several adverse health effects of chronic exposure to selenium, of which an overdose affected endocrine and neurological function. They also stated that chronic exposure to selenium causes hepatotoxicity and gastrointestinal disturbances. Based on the in vitro study, selenium is toxic to human hepatocellular carcinoma cells. A prospective, placebo-controlled, randomized, double-blind, phase II study shows that patients under intensive care who received sodium selenite (4000 μg on day-1 and 1000 μg daily for another nine days) survived from the chronic exposure to selenium [107]. As many studies reported on the health benefits of selenium compared to the adverse effects [108], selenium toxicity is, therefore, not a serious matter.

## 7. Conclusions

Vitamin A, lutein, and zeaxanthin are potent components in the retina of the human eye. Although many studies reported the protective effects of these compounds against AMD and other eye diseases, some of the clinical trials and epidemiological studies reported negative findings on the prevention of AMD. In most of the studies, vitamin and mineral supplementations failed to significantly improve eye condition and did not effectively reduced the risk of AMD. However, vitamins C and E, as well as selenium, act as antioxidants in reducing the cellular oxidative stress of the retina or macular region of the eye. Anthocyanins have positive effects on AMD, and—based on in vitro and animal models—has only one study with a negative finding on anthocyanins reducing the risk of AMD. Based on human studies, dietary carotenoids demonstrated positive results, except for two studies. One of the studies showed no significant effect of total daily oral supplementation of β-carotene and other vitamins on the secondary outcomes of AMD compared to placebo, and another study reported no significant increase in macular pigment optical density in postmenopausal women that were supplemented with lutein and zeaxanthin daily. Controversial results have been reported by the researchers who performed human studies on vitamin supplementation and prevention or risk reduction of AMD, where four out of ten findings are favorable. One negative finding has been reported previously on the prevention of AMD by either zinc or selenium. Four human studies also showed that zinc supplementation effectively reduced the risk of AMD and visual loss. More studies should be focused on specific formulas with a combination of these antioxidants for prevention of its early development and the treatment of macular degeneration and other eye-related complications which including cataract, glaucoma, and night blindness. Supplementation of these nutrients should be in moderate doses following evidence-based recommended dosages and reference intakes and are advisable to be taken as a dietary supplement. Future studies should be focused on the determination of optimal doses of anthocyanins and carotenoids for reducing the risk of AMD, as well as the toxic doses of these natural occurring phytochemicals. Due to the toxic effect of synthetic compounds, dietary supplements from natural sources warrant the safeness of consuming these antioxidants for better health.

## Figures and Tables

**Table 1:** Protective effects of anthocyanins and carotenoids against age-related macular degeneration and eye-related complications.

Compounds	Study Design	Doses	Outcomes	Ref.
*Anthocyanins*				
Cyanidin 3-glucoside, cyanidin 3-rutinoside, delphinidin 3-glucoside, and delphinidin 3-rutinoside	In vitro bioassays: Rod outer segment and opsin membranes of frog	10–50 µM	Positive outcomes: Cyanidin 3-glucoside and cyanidin 3-rutinoside stimulated regeneration of rhodopsin	[21]
Cyanidin 3-glucoside and delphinidin 3-glucoside	Cell culture: ARPE-19 cells (human retinal pigment epithelial cell line)	5 μM	Positive outcome: Anthocyanins pre-treatment attenuated apoptosis of ARPE-19 cells induced by UVB irradiation.	[23]
Bilberry anthocyanin extract	In vivo study: Retinal degeneration model in pigmented rabbits (seven days)	250 and 500 mg/kg/day	Positive outcomes: Attenuated changes caused by light to Bax, Bcl-2, and caspase-3. Increased the levels of superoxide dismutase, glutathione peroxidase, catalase, and total antioxidant capacity. Decreased malondialdehyde level in the retinal cells. Inhibited light-induced elevation in the levels of pro-inflammatory cytokines and angiogenic parameters (IL-1β and VEGF).	[22]
Anthocyanin supplement	Randomized, parallel study. Postmenopausal, one woman (eight months)	60 mg/day	Negative outcome (compared to baseline): No significant increase in macular pigment optical density	[26]
*Carotenoids*				
Lutein and zeaxanthin	Cell culture: ARPE-19 cells	5 μM	Positive outcome: Anthocyanins pre-treatment attenuated apoptosis of ARPE-19 cells induced by UVB irradiation.	[23]
Lutein and zeaxanthin	Prospective, randomized, double-blind, placebo-controlled human study (12 months)	10 mg/day lutein and 2 mg/day zeaxanthin	Positive outcomes: Significantly increased macular pigment optical density for treatment group compared to placebo. Significantly increased levels of serum lutein and zeaxanthin. Significantly improved chromatic contrast and photo stress recovery time for treatment group compared to placebo.	[10]
Zeaxanthin-containing spirulina (4–5 g)	Human feeding trials (45 days)	2.6–3.7 mg zeaxanthin	Positive outcome: Increased mean serum zeaxanthin concentration from 0.06 to 0.15 μmol/L.	[11]
Lutein, zeaxanthin, and meso-zeaxanthin in sunflower oil suspension	Double-blind, placebo-controlled, block-randomized human trial (12 months)	10 mg lutein, 10 mg meso-zeaxanthin, and 2 mg zeaxanthin	Positive outcomes: Significantly improved contrast sensitivity of the visual function after 12 months supplementation compared to baseline. Treatment group had significant increase in serum concentrations of the xanthophylls in retina and macular pigment optical density compared to placebo.	[12]
Lutein vs. α-tocopherol	Randomized, double-blind, placebo-controlled supplementation study (24 months)	12 mg lutein mixtures and 100 mg α-tocopherol	Positive outcomes: Significantly increased serum concentration of lutein. Increased visual performance (visual acuity and glare sensitivity) in lutein group only. No toxic effect found—no significant changes in hematological and biochemical profiles.	[30]
Oral total daily supplementation of antioxidants (mixture of β-carotene with other vitamins)	Randomized, placebo-controlled clinical trial (followed up for up to 10 years)	15 mg β-carotene	Positive primary outcome (compared to baseline): Reduced risk of visual acuity lost. Negative secondary outcomes: No significant differences for all the secondary outcomes between the treatment group and placebo.	[31]
Nutrient intake (β-carotene, β-cryptoxathin, lutein, zeaxanthin, and lycopene)	Epidemiological study (Self-report data)	-	Positive outcome: Participants with the highest self-reported dietary intake of lutein and zeaxanthin were inversely associated with advancedage-related macular degeneration (AMD).	[37]
Total carotenoids (lutein/zeaxanthin, α-carotene, β-carotene, cryptoxanthin, and lycopene	Eye Disease Case-Control Study	-	Positive outcome: Serum carotenoid level significantly associated with the risk of AMD	[38]
Xanthophyll supplement	Randomized, parallel study. Postmenopausal women (8 months)	6 mg lutein and 2 mg zeaxanthin daily)	Positive outcome: Dietary supplementation of lutein and zeaxanthin significantly increased the serum lutein and zeaxanthin levels. Negative outcome (compared to baseline): No significant increase in macular pigment optical density	[26]

**Table 2:** Positive and negative outcomes of vitamins and minerals against age-related macular degeneration and eye-related complications.

Compounds	Study Design	Doses	Outcomes	Ref.
*Vitamins*				
Mixture of vitamin C and vitamin E with provitamin A carotenoid	Randomized, placebo-controlled clinical trials (followed-up for up to 10 years)	Vitamin C (500 mg) and vitamin E (400 IU) daily	Positive primary outcomes (compared to baseline): Increase in nuclear, cortical, or posterior subcapsular opacity grades or cataract surgery. Moderate visual acuity lost (≥15 letters). Negative secondary outcomes: No significant differences for all the secondary outcomes between the treatment group and placebo.	[11]
Provitamin A β-carotene, vitamin C, and vitamin E	Age-Related Eye Disease Study	-	Positive outcomes: Increased intake of β-carotene, vitamin C, and vitamin E associated with a reduced risk of neovascular AMD.	[42]
Vitamin A, vitamin C, and vitamin E	Systematic review and meta-analysis	-	Positive outcomes: Dietary intake of a mixture of vitamin A, vitamin C, and vitamin E had a larger effect on the reduction of AMD risk than the individual vitamin.	[43]
Vitamin A, vitamin C, and vitamin E	Case-control study	-	Positive outcomes: Low dietary intake of vitamin C and vitamin E was associated with neovascular AMD. Negative outcome: Dietary vitamin A showed no association with neovascular AMD.	[58]
Vitamin E	Randomized controlled trial (four years)	500 IU daily	Negative outcomes: Failed to prevent the development and progression of AMD.	[55]
Vitamin E	Randomized placebo controlled 4-arm trial (follow-up of 5.6 ± 1.2 years)	400 IU daily (DL-α-tocopherol acetate)	Negative outcome: Vitamin supplementation showed no protective effect against cataracts among the participants (elderly men).	[57]
Vitamin A, vitamin C, and vitamin E	Multicenter eye disease case-control study (Epidemiological study)	-	Negative outcomes: Vitamins A, C, and E consumptions were not associated with the reduced risk of AMD.	[39]
Vitamin A (retinol), vitamin C (ascorbic acid), and vitamin E (α-tocopherol)	POLA (Pathologies Oculaires Liées à l’Age) study	-	Negative outcomes: Plasma vitamin A and vitamin C showed no association with reduction in macular degeneration risk. Plasma vitamin E was negatively associated with early signs of AMD and late AMD.	[40]
Vitamin C	Cochrane Review	-	Negative outcomes: Vitamin C supplementation did not prevent any AMD or late AMD.	[46]
Vitamin C and vitamin E	Eye Disease Case-Control Study	-	Negative outcome: No statistically significant overall association was found between serum vitamin status and neovascular AMD.	[47]
*Minerals*				
Zinc	Case-control study	-	Positive outcome: Low dietary intake of zinc was associated with neovascular AMD.	[58]
Zinc	Randomized, placebo-controlled clinical trials (followed-up for up to 10 years)	Zinc oxide (80 mg daily)	Positive outcome: Significantly reduced the risk of developing advanced AMD.	[11]
Zinc	Randomized double-blinded, placebo-controlled trials (2 years intervention)	Zinc sulfate (200 mg daily)	Positive outcome: Significantly reduced visual loss in treatment group compared to placebo.	[59]
Zinc	Randomized, prospective, placebo-controlled clinical trial (three and six months intervention)	Zinc monocysteine (25 mg daily)	Positive outcomes: Significantly improved visual acuity and contrast sensitivity. Significantly shortened macular light flash recovery time both at three months and at six months.	[60]
Zinc	Randomized, double-blinded, placebo-controlled study (two years intervention)	Zinc sulfate (200 mg daily)	Positive outcome: Significantly increased serum zinc. Negative outcome: No significant improvement of eye conditions for patients with AMD.	[61]
Selenium	Randomized, placebo-controlled, 4-arm trial (follow-up of 5.6 ± 1.2 years)	200 μg daily (from l-selenomethionine)	Negative outcome: Selenium supplementation did not show significant effect in reducing risk of cataracts among the participants (elderly men).	[57]

**Table 3:** Recommended daily intakes and tolerable upper intake level (UL) for vitamins and minerals [88].

Nutrients	Recommended Daily Intake	UL
*Vitamins*		
Vitamin A	700 μg RE/day for men and 600 μg RE/day for women	3000 μg RE/day
Vitamin C	<1000 mg/day	1000 mg/day
Vitamin E	300 mg α-tocopherol equivalents (450 IU)	300 mg α-tocopherol equivalents (450 IU)
*Minerals*		
Selenium	26–55 µg/day	300 μg/day
Zinc	11 mg/day for men and 8 mg/day for women	25 mg/day
